# Neural fingerprinting on MEG time series using MiniRocket

**DOI:** 10.3389/fnins.2023.1229371

**Published:** 2023-09-20

**Authors:** Nikolas Kampel, Christian M. Kiefer, N. Jon Shah, Irene Neuner, Jürgen Dammers

**Affiliations:** ^1^Institute of Neuroscience and Medicine (INM-4), Forschungszentrum Jülich GmbH, Jülich, Germany; ^2^Faculty of Medicine, RWTH Aachen University, Aachen, Germany; ^3^Jülich Aachen Research Alliance (JARA) – CSD – Center for Simulation and Data Science, Aachen, Germany; ^4^Faculty of Mathematics, Computer Science and Natural Sciences, RWTH Aachen University, Aachen, Germany; ^5^Jülich Aachen Research Alliance (JARA) – BRAIN – Translational Medicine, Aachen, Germany; ^6^Institute of Neuroscience and Medicine (INM-11), Jülich Aachen Research Alliance (JARA), Forschungszentrum Jülich GmbH, Jülich, Germany; ^7^Department of Neurology, University Hospital RWTH Aachen, Aachen, Germany; ^8^Department of Psychiatry, Psychotherapy and Psychosomatics, RWTH Aachen University, Aachen, Germany

**Keywords:** neural fingerprinting, resting state, rocket, time series classification, magnetoencephalogra, MEG, machine learning

## Abstract

Neural fingerprinting is the identification of individuals in a cohort based on neuroimaging recordings of brain activity. In magneto- and electroencephalography (M/EEG), it is common practice to use second-order statistical measures, such as correlation or connectivity matrices, when neural fingerprinting is performed. These measures or features typically require coupling between signal channels and often ignore the individual temporal dynamics. In this study, we show that, following recent advances in multivariate time series classification, such as the development of the RandOm Convolutional KErnel Transformation (ROCKET) classifier, it is possible to perform classification directly on short time segments from MEG resting-state recordings with remarkably high classification accuracies. In a cohort of 124 subjects, it was possible to assign windows of time series of 1 s in duration to the correct subject with above 99% accuracy. The achieved accuracies are vastly superior to those of previous methods while simultaneously requiring considerably shorter time segments.

## 1. Introduction

Historically, neuroscientists have inferred knowledge about the brain from the population level, and commonalities between individuals were used as the foundation for our understanding of the brain (van Horn et al., 2008). However, it is now known that individual variations may convey important information, and disregarding them as noise may limit our insight into the brain [see (van Horn et al., 2008) for a review]. Placing the individual as the focus of research led to the emergence of the field of neural fingerprinting, i.e., the identification of individuals in a cohort using different neuroimaging modalities such as magnetic resonance imaging (Wachinger et al., 2015; Valizadeh et al., 2018), functional magnetic resonance imaging (Miranda-Dominguez et al., 2014; Finn et al., 2015; Kaufmann et al., 2017; Amico and Goñi, 2018; Bari et al., 2019), functional near-infrared spectroscopy (de Souza Rodrigues et al., 2019), electroencephalography (Rocca et al., 2014; Fraschini et al., 2015; Kong et al., 2019), and magnetoencephalography (MEG) (da Silva Castanheira et al., 2021; Sareen et al., 2021).

The development of neuroimaging techniques has further led to the possibility of using second-order statistical summaries of brain activity, such as functional connectomes, as the basis for neural fingerprinting (Sareen et al., 2021). However, functional connectomes are not necessarily required for neural fingerprinting as neural fingerprinting can be performed directly on the time series from which the connectomes are usually computed. In fact, (multivariate) time series classification ((M)TSC), where unlabeled time series are assigned to one of multiple classes, is an exciting, yet challenging, field of research (Keogh and Kasetty, 2003; Yang and Wu, 2006). For example, many practical applications have emerged for (M)TSC in fields such as biology, medicine, finance, or engineering (Keogh and Kasetty, 2003). Despite these advances, applications have been limited due to the fact that time series classification methods are computationally expensive (Abanda et al., 2019).

Recently, a fast approach for time series classification, known as RandOmConvolutionalKErnelTransform (ROCKET), has been introduced and requires only a fraction of the computational expense of most existing methods (Dempster et al., 2020). Its new variant, called MiniRocket (MINImally RandOm Convolutional KErnel Transform), introduced by the same group, provides similar or better accuracy but is up to 75 times faster compared to ROCKET on larger datasets (Dempster et al., 2021).

Given these capabilities, we sought to reduce the complexity of neural fingerprinting by directly applying the multivariate time series classifier MiniRocket to source time courses from MEG resting-state recordings. Data requirements for training a successful classifier were investigated. Furthermore, it has been suggested that day-to-day variations in the background noise may have a significant impact on the classification results (da Silva Castanheira et al., 2021). Therefore, we conducted experiments to estimate the effect of background noise by incorporating empty-room recordings (i.e., noise recordings taken without a subject being measured) into the training and testing datasets.

Using MiniRocket, it was possible to differentiate between MEG resting-state recordings from 124 subjects with accuracies exceeding 99.5%. A set of parameters providing a good trade-off between accuracy, speed, and amount of available data was investigated. Based on our findings, the impact of background noise on the classification results for fingerprinting appears to be minimal.

## 2. Methods

### 2.1. Time series classification

In a similar way to image classification, TSC also requires the input values to be ordered, and it is possible that important information relevant to the classification might be buried in the ordering process (Bagnall et al., 2017). Moreover, in the case of a multivariate time series, discriminatory features might even depend on interactions between the individual time series, and special multivariate classifiers are needed to deal with this added complexity (Ruiz et al., 2021). While it is generally possible to adapt strictly univariate classifiers to the multivariate case, for example, by using an ensemble of separate univariate classifiers for each of the multivariate dimensions, inter-dimensional dependencies are ignored, and information is inevitably lost (Ruiz et al., 2021).

A variety of MTSC methods, which include ensembles of univariate classifiers such as Hierarchical Vote Collective of Transformation-based Ensembles (HIVE-COTE) (Bagnall et al., 2020), dedicated multivariate TSC methods such as RandOm Convolutional KErnel Transformation (ROCKET) (Dempster et al., 2020), MINImally RandOm Convolutional KErnel Transform [MiniRocket, (Dempster et al., 2021)] and deep-learning approaches such as InceptionTime (Ismail Fawaz et al., 2020), were recently reviewed for their performance on openly available TSC datasets (Ruiz et al., 2021). Due to the exceptionally fast training times and state-of-the-art classification accuracy, we elected to use MiniRocket in this paper.

#### 2.1.1. Rocket

The basic principle behind ROCKET is to randomly generate a large number of convolutional kernels, which are then applied to the multivariate time series to obtain transformed features. Finally, a linear classifier, such as logistic regression or ridge regression, is trained on the transformed ROCKET features (Dempster et al., 2020). Since the training complexity is linear in both the length of the time series and the number of training samples, ROCKET is an attractive, scalable algorithm for large datasets (Dempster et al., 2020).

There are five basic parameters that characterize a random convolutional ROCKET kernel: length, *l_k_* and dilation, *d*, the individual weights, *w*, a bias term, *b*, and the use of padding (Ismail Fawaz et al., 2019; Dempster et al., 2020). The convolution, *C*, of the ROCKET kernel with a univariate time series can be computed by performing a sliding dot product operation over time *t* across the entire time series:


(1) $$C_{t}=X_{t}*w+b=\left(\overset{l_{k}-1}{\underset{j=0}{\sum }}X_{t+\left(j\times d\right)}w_{j}\times w_{j}\right)+b.$$

Since patterns in the time series congruent with the kernel will result in large values (Ismail Fawaz et al., 2019; Dempster et al., 2020), basic patterns or shapes can thus be detected. In ROCKET, global max pooling and the proportion of positive values (ppv) pooling are applied separately to the kernel output, providing two features per kernel. By using ppv pooling, ROCKET weights the prevalence of a feature captured by the kernel output over *n* time samples, *t*.


(2) $$\mathrm{ppv}=\frac{1}{n}\overset{n-1}{\underset{i=0}{\sum }}\left[C_{i}>0\right].$$

By using different values for the dilation, it is possible to capture patterns at different scales, and it is even possible to capture frequency information with larger dilation values corresponding to smaller frequencies and vice versa (Yu and Koltun, 2016).

ROCKET generates the kernel parameters based on several predefined rules. First, the length of a kernel is selected with uniform probability from the set {7, 9, 11}. Then, the weights are sampled from a normal distribution, *w_j_N*(0,1), and subsequently mean centered, i.e., after all weights have been determined, the mean weight is subtracted. A uniform distribution is used to sample the bias term with *bU*(−1,1). The dilation is sampled from an exponential scale with *d* = [2*^x^*] where *xU*(0, *A*) and *A* = log_2_(*l*_input_^−1^/*l_k_*^−1^). Finally, a binary decision with equal probability determines whether padding is used, i.e., whether (*l_k_* − 1)/2 zeros are added to the beginning and the end of the time series (Dempster et al., 2020).

For multivariate time series, an additional sixth kernel parameter is provided, which determines the particular dimensions a given kernel is applied to Ruiz et al. (2021). The kernels then become matrices with independently generated weights for each dimension, and consequently, the convolution is computed as the sliding dot product between two matrices (Ruiz et al., 2021).

The feature that makes ROCKET special, and distinguishes it from earlier methods using (random) convolutional kernels, is the huge number and variety of kernels (10,000 per default) (Dempster et al., 2020). Furthermore, a key contributor to the ability of ROCKET to detect patterns at different scales and frequencies is its effective use of dilation (Dempster et al., 2020). Yet, the potentially most important aspect of ROCKET’s success is that ROCKET computes two features for each kernel: the maximum value (similar to global max pooling) and a novel feature called the proportion of positive values, which provides the classifier with information about the prevalence of a given pattern in the time series (Dempster et al., 2020). Thus, the use of effective features and the combination of a large number of kernels enable ROCKET to distinguish between a multitude of time series patterns for the purpose of classification.

Finally, the ROCKET features are used to train a linear classifier. Logistic regression with stochastic gradient descent was recommended for very large datasets where the number of training examples is significantly higher than the number of features while, for smaller datasets, the authors recommended the use of ridge regression with cross-validation for the regularization parameter (Dempster et al., 2020).

#### 2.1.2. MiniRocket

The major difference between MiniRocket and ROCKET is that it uses a fixed set of convolutional kernels instead of kernels with random hyperparameters. In brief, the kernel length, *l_k_* in MiniRocket is fixed to 9 instead of {7, 9, 11}, and the kernel weights are restricted to either −1 or 2 instead of a weight drawn from a normal distribution between 0 and 1. Moreover, MiniRocket uses fixed padding, and the maximum number of dilation per kernel is restricted to 32 (Dempster et al., 2021). These features allow the method to minimize the number of hyperparameters per kernel, enabling faster computation. Moreover, MiniRocket computes the kernel weights, *w* and −*w* and the ppv at the same time by using a trick: with the proportion of negative values being pnv = 1 − ppv, MiniRocket uses the ppv of the inverted kernel without increasing the number of convolutions, thus doubling the number of kernels applied using a single convolution. In addition, several mathematical optimizations are applied [for details, see (Dempster et al., 2021)] that makes MiniRocket much faster (up to 75 times) compared to ROCKET, while maintaining the same accuracy (Dempster et al., 2021).

### 2.2. The data

MEG recordings from two different sites (United States and Germany) were used for analysis. The first dataset was obtained from the Human Connectome Project (HCP), while the second dataset was provided by the Institute of Neuroscience and Medicine at Forschungszentrum Jülich (FZJ), Germany. MEG data in the two datasets were recorded at various points in time. For each subject, a minimum of two resting-state measurements and at least one empty-room recording were available. The total number of MEG recordings used was 372 from 124 different subjects.

#### 2.2.1. Dataset HCP

The Human Connectome Project (HCP) offers open access to a dataset consisting of MEG resting-state recordings and anatomical MR scans for 89 subjects acquired at St. Louis University (Van Essen et al., 2012, 2013; Larson-Prior et al., 2013; Hodge et al., 2016). From this dataset, we used recordings from 84 subjects, 44% of whom were female, and the mean age was 28.9 ± 3.6 years. Between two and three resting-state recordings with durations of approximately 6 min were available for each subject. Furthermore, an empty-room measurement of approximately 5 min in duration was available for each subject.

All MEG data were acquired using a whole-head MAGNES 3600 system (4D Neuroimaging, San Diego, CA) with 248 magnetometers and 23 reference channels at a sampling rate of 2034 Hz. ECG and EOG were acquired along with the MEG signals. At the beginning of each MEG recording session, the subject’s head shape, together with the positions of the localizer coils, were digitized for the alignment with the anatomical MR scans, which were recorded as T1-weighted volumes with 0.7 mm resolution using a Skyra 3 T scanner (Siemens Healthcare GmbH, Erlangen, Germany).

#### 2.2.2. Dataset FZJ

The FZJ dataset consists of two different MEG resting-state recording sessions. The first one was acquired from 20 male subjects in 2012 and 2013, and the second set was acquired from another set of 20 subjects (55% female) in 2017 and 2018. The mean ages were 26.2+/− 4.3 and 26.6+/− 4.9 years, respectively. While the recordings from 2012 and 2013 had a duration of approximately 3 min, followed by empty room recordings of about 5 min, the recordings from 2017 and 2018 had a duration of 6 min, followed by empty room recordings of between 10 and 15 min. Similar to the HCP data, a whole-head MAGNES 3600 system with 248 magnetometers and 23 reference channels was used; however, the sampling rate was 1017.25 Hz.

Electrocardiography (ECG) and electrooculography (EOG) were recorded using the MAGNES 3600 system along with the MEG measurements. An external BrainAmp ExG system (Brain Products, Gilching, Germany) was used to record ECG and EOG at a sampling rate of 5,000 Hz for the later recordings (2017 and 2018). The subjects’ head shapes were digitized prior to the MEG recording sessions for alignment with the anatomical MR scans, which were recorded using a MAGNETOM 3 T scanner (Siemens, Munich, Germany) with MPRAGE (Mugler and Brookeman, 1990).

### 2.3. Data analysis

Python 3.10 was used for data analysis, with the main packages being MNE-Python v1.3.1 (Gramfort et al., 2013, 2014), Scikit-learn v1.2.2 (Pedregosa et al., 2011), and sktime v0.17.1 (Löning et al., 2019). The source spaces were constructed from the anatomical MR scans based on an octahedral mesh using FreeSurfer (Dale et al., 1999; Fischl et al., 1999).

#### 2.3.1. Pre-processing

The first step in the pre-processing pipeline was to identify MEG channels with strong artifacts. An in-house machine learning algorithm based on density-based spatial clustering of applications with noise (DBSCAN) (Ester et al., 1996), which scans for artifacts both in the time and the frequency domain, was used for this purpose. Channels and time segments with strong artifacts were annotated as ‘bad’ and were followed by a visual inspection of the automated procedure. Furthermore, all recordings were also visually inspected for segments containing unusually strong artifacts (e.g., muscle artifacts), which were discarded from the analysis. The signals of the annotated bad channels were subsequently replaced by virtual channels using the interpolation method as implemented in (Gramfort et al., 2013, 2014). Table 1 summarizes the duration of the MEG recordings used for each dataset and the recording type (resting-state or empty room data).

**Table 1:** Median recording times and its ranges for the type of recording after the removal of bad data segments.

Dataset	Rec. type	*T*_median_	*T*_max_	*T*_min_
FZJ	Empty	460	911	271
FZJ	rs1	220	299	136
FZJ	rs2	231	298	151
HCP	Empty	275	300	171
HCP	rs1	291	300	243
HCP	rs2	293	300	232

Times (T) in seconds.

Next, the MEG signals were band-pass filtered from 1 to 200 Hz. Environmental and power line noise was removed by subtraction of appropriate weighted reference signals from the band-pass filtered (0.1 to 5 Hz) references signals as described in (Robinson, 1989). Furthermore, power-line noise (50 Hz in Germany and 60 Hz in the United States of America) plus harmonics were isolated in the reference channels using anti-notch filters at these frequencies. The weighted signal from the reference channels was then subtracted from the signal channels to reduce power-line noise.

Finally, ECG and EOG artifacts were removed using independent component analysis (ICA) (Hyvärinen and Oja, 2000; Dammers et al., 2008). Components containing significant contributions of cardiac or ocular activity were removed prior to source localization (Hyvärinen and Oja, 2000; Dammers et al., 2008).

#### 2.3.2. Source localization and extraction of label time courses

The pre-processed, continuous MEG resting-state signals were projected onto the source space using the minimum-norm estimate (MNE) method (Hämäläinen and Ilmoniemi, 1994). The source spaces were then divided into 68 (34 per hemisphere) anatomical regions (labels) based on the Desikan-Killiany Atlas (Desikan et al., 2006). As the frontal pole region is very small in this particular atlas, the number of vertices identified was very small, and no vertices were found in this region for one subject. Therefore, this subject was excluded from the analysis. Following this step, a single representative source time course was extracted for each region as the mean time course of all vertices inside this brain region. Finally, these continuous source time courses were split into time segments of different lengths (hereafter referred to as ‘trials’).

The same pre-processing and source localization steps were repeated for the empty-room data, with the data being treated as if it were a subject’s recording. The empty-room data, which contain environmental noise only, are recorded directly after the MEG recordings. To further investigate whether day-to-day environmental noise variability causes significant differences, all empty-room recordings were also projected onto the same source space of a randomly selected subject. In this way, the influence of the background noise can be minimized, allowing the classifier to use the recordings for fingerprinting decisions.

### 2.4. Classification

sktime (version 0.17.1) was used to perform the MiniRocket transformation of the MEG trials, and scikit-learn (version 1.2.2) was used to fit a ridge regression classifier to the transformed features.

To evaluate the classification performance, we compute the accuracy (ACC) as the ratio of the number of correctly classified instances to the total number of instances. In relation to neural fingerprinting, we test how accurately the model detects whether two different datasets from the same subject match. In addition to the ACC, the Precision, the Recall, and the F1-Score are computed.

The Precision refers to the proportion of correctly predicted positive instances out of all the instances predicted as positive by the model and is defined by Precision = TP/(TP + FP), with TP and FP being the True Positive and False Positives, respectively. A high precision value indicates that the model has a low rate of false positives. Recall (a.k.a. Sensitivity) is defined by Recall = TP/(TP + FN), with FN being the False Negatives, and measures the proportion of actual positive instances that are correctly identified by the model. Higher Recall indicates that the model is better at identifying all relevant positive instances in the dataset. The F1-Score is defined by F1-Score = 2 * (Precision * Recall)/(Precision + Recall). Thus, the F1-Score provides a balance between Precision and Recall and ranges from 0 to 1, where 1 represents perfect precision and recall, and 0 indicates poor performance. We report the macro-average F1-Score, Precision, and Recall for each class independently and then take the average across all classes to ensure that the performance of each class (the subject) is given equal importance.

To evaluate the overall performance of the model, we employed a leave-one-out method (LOOM) at the subject level (Schlögl and Supp, 2006). Specifically, each subject was left out of the training and test sets once. This results in a total of 124 mean scores (e.g., accuracy) for each of the two training and test variants, for which the overall mean and standard deviation are computed. In this way, the stability of the model performance and the influence of data from individuals can be evaluated by computing the variance of the performance metrics.

#### 2.4.1. Resting-state neural fingerprinting

To investigate the performance of the classifier with respect to identifying a specific subject within the cohort, time series originating from the first resting-state recording (rs1) were used for training, while time series originating from the second resting-state recording (rs2) were used for testing. This order was then reversed to determine a broader estimate of the classifier’s performance.

The continuous source time course of each brain region was used for a *z*-scored normalization. A random but fixed subset of trials was sampled from each recording to ensure balanced datasets across subjects. To gauge the variance expected due to the random nature of the method, we repeated the procedure ten times using random selections of trials and kernel initializations. The classifier’s dependence on several parameters was tested by means of varying the number of trials used per subject in the training set, the trial duration, and the number of ROCKET kernels used.

#### 2.4.2. Empty-room noise

To assess the impact of the day-to-day variations in the background noise with respect to the classification performance, we performed a control experiment with identical settings but with no subject in the scanner. These so-called empty room recordings were performed directly after the subject recording and were labeled with the same ID as the subject. In other words, the environmental noise data is used to have a third control condition to evaluate the model. With the empty-room noise data as a third set of recordings (rs1, rs2, empty), we performed the training and the testing of the model for all possible combinations. Each experiment was repeated ten times with a random selection of trials as well as different random kernel initializations. The mean accuracy was computed for each combination.

## 3. Results

### 3.1. Resting-state neural fingerprinting and its dependency on parameters

The classification of two MEG datasets recorded from the same subjects on the same day revealed remarkably high accuracy scores of about 99% using MiniRocket. The impact of important parameters on the classification accuracy was tested by varying the number of kernels, the number of trials, and the trial duration. While investigating the impact of one parameter, all other parameters were fixed as follows (unless stated otherwise): the number of kernels was set to 3,500, the number of trials to 15, and its duration to 1.5 s.

**Figure 1:**
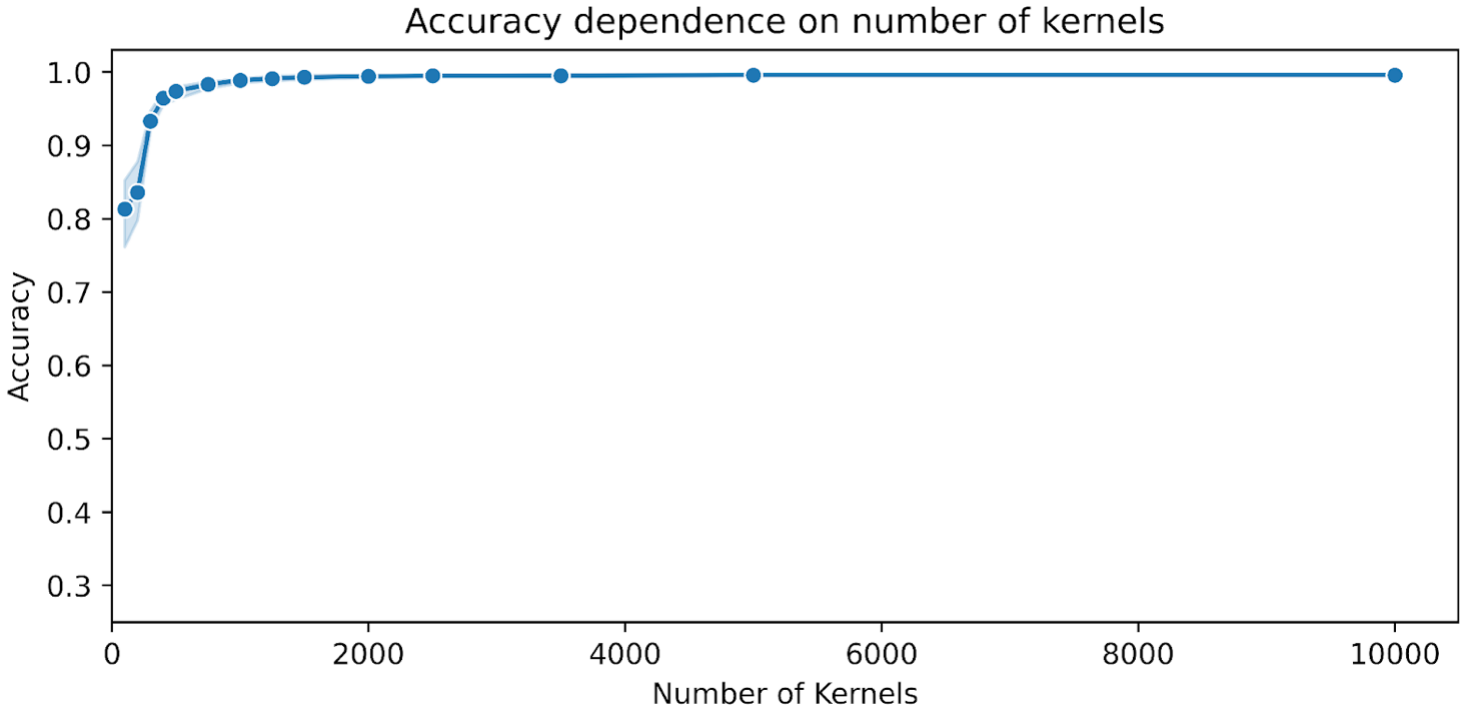
Dependence of the accuracy scores on the number of MiniRocket kernels. The classifier was trained on 15 time segments with a duration of 1.5 s per segment. The blue shaded area indicates the upper and lower range of the classification accuracy between the 10 repetitions with a random selection of time segments and a random initialization of kernels.

Figure 1 shows the dependency of the accuracy scores on the number of kernels used in MiniRocket. The figure shows a sharp increase in accuracy between 100 and 500 kernels, with scores already above 96% for 500 kernels. For the number of kernels ranging from 1,000 to 5,000, there was a relatively marginal increase in accuracy, which only ranged from about 98.9 to 99.5%. All results, including the upper and lower range, can be found in Table 2.

**Table 2:** MiniRocket accuracy scores dependent on several parameters.

Dependence on number of kernels (15 training trials, 1.5 s duration)			
Number of kernels	Mean accuracy	Min accuracy	Max accuracy
100	81.32	76.16	85.27
200	83.6	79.78	87.8
300	93.33	91.96	94.73
400	96.45	95.51	97.34
500	97.37	96.26	98.01
750	98.32	97.74	98.71
1,000	98.87	98.52	99.06
1,250	99.11	98.79	99.52
1,500	99.3	98.98	99.6
2000	99.42	99.22	99.6
2,500	99.49	99.3	99.57
3,500	99.51	99.3	99.7
5,000	99.57	99.49	99.65
10,000	99.59	99.49	99.65

Dependence number of trials (3,500 kernels, trial duration 1.5)			
Number of trials	Mean accuracy	Min accuracy	Max accuracy
1	63.63	56.45	70.16
2	90.18	86.29	93.15
3	96.36	94.76	97.58
4	97.97	97.48	98.29
5	98.63	98.31	98.87
6	98.97	98.66	99.26
7	99.11	98.85	99.37
8	99.22	98.94	99.5
9	99.31	99.06	99.55
10	99.35	99.15	99.52
12	99.45	99.13	99.56
15	99.51	99.3	99.7
20	99.57	99.54	99.6
25	99.58	99.56	99.61

Dependence on trail duration (3,500 kernels, 15 training trails)			
Trail duration	Mean accuracy	Min accuracy	Max accuracy
0.1	29.56	26.02	31.77
0.2	71.15	67.66	73.76
0.3	89.64	88.12	91.64
0.4	95.67	95.22	96.26
0.5	97.88	97.58	98.28
0.6	98.58	98.2	99.03
0.7	99.01	98.79	99.33
0.8	99.2	98.87	99.46
0.9	99.28	99.11	99.52
1.0	99.37	99.09	99.6
1.25	99.5	99.33	99.57
1.5	99.51	99.3	99.7
2.0	99.55	99.33	99.65
2.5	99.58	99.49	99.62
3.0	99.6	99.57	99.62
3.5	99.6	99.57	99.68
4.0	99.6	99.54	99.68
4.5	99.61	99.57	99.7
5.0	99.6	99.57	99.68

**Figure 2:**
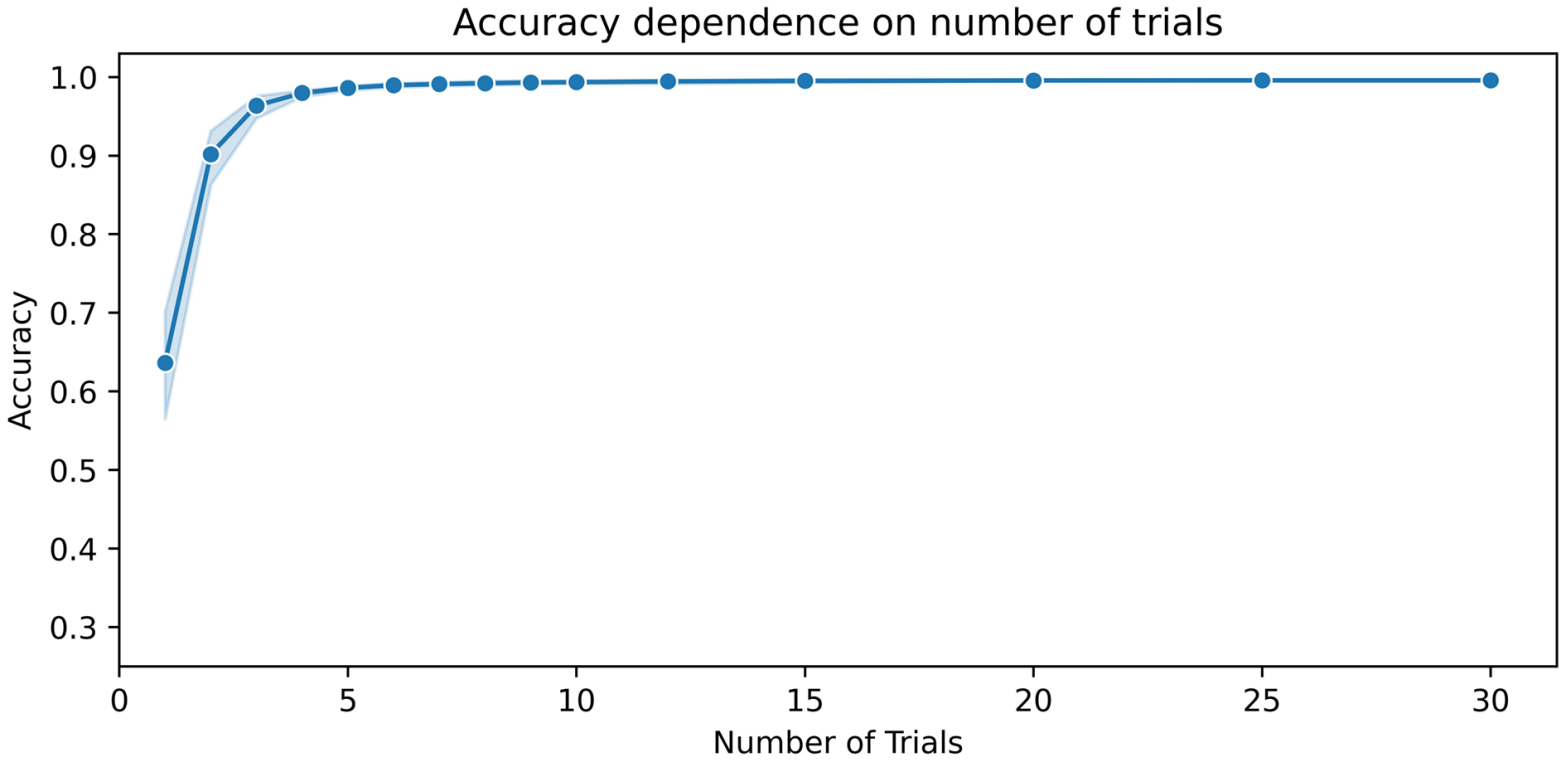
Dependence of the accuracy scores on the number of training segments. The number of kernels for the MiniRocket classifier was set to 3,500. The duration of the time segments was set to 1.5 s. The blue shaded area indicates the upper and lower range of the classification accuracy between the 10 repetitions with a random selection of time segments and a random initialization of kernels.

To estimate the impact of the number of time segments used on the classification result, the number of trials was gradually increased until no further change in accuracy was observed. Figure 2 shows the dependence of accuracy scores on the number of training trials. The figure shows that when five or more trials are used, classification accuracies of 98% and above can be achieved. Only a marginal increase in accuracy, ranging from about 99.3 to 99.6%, was achieved from 10 to 30 trials (Table 2).

**Figure 3:**
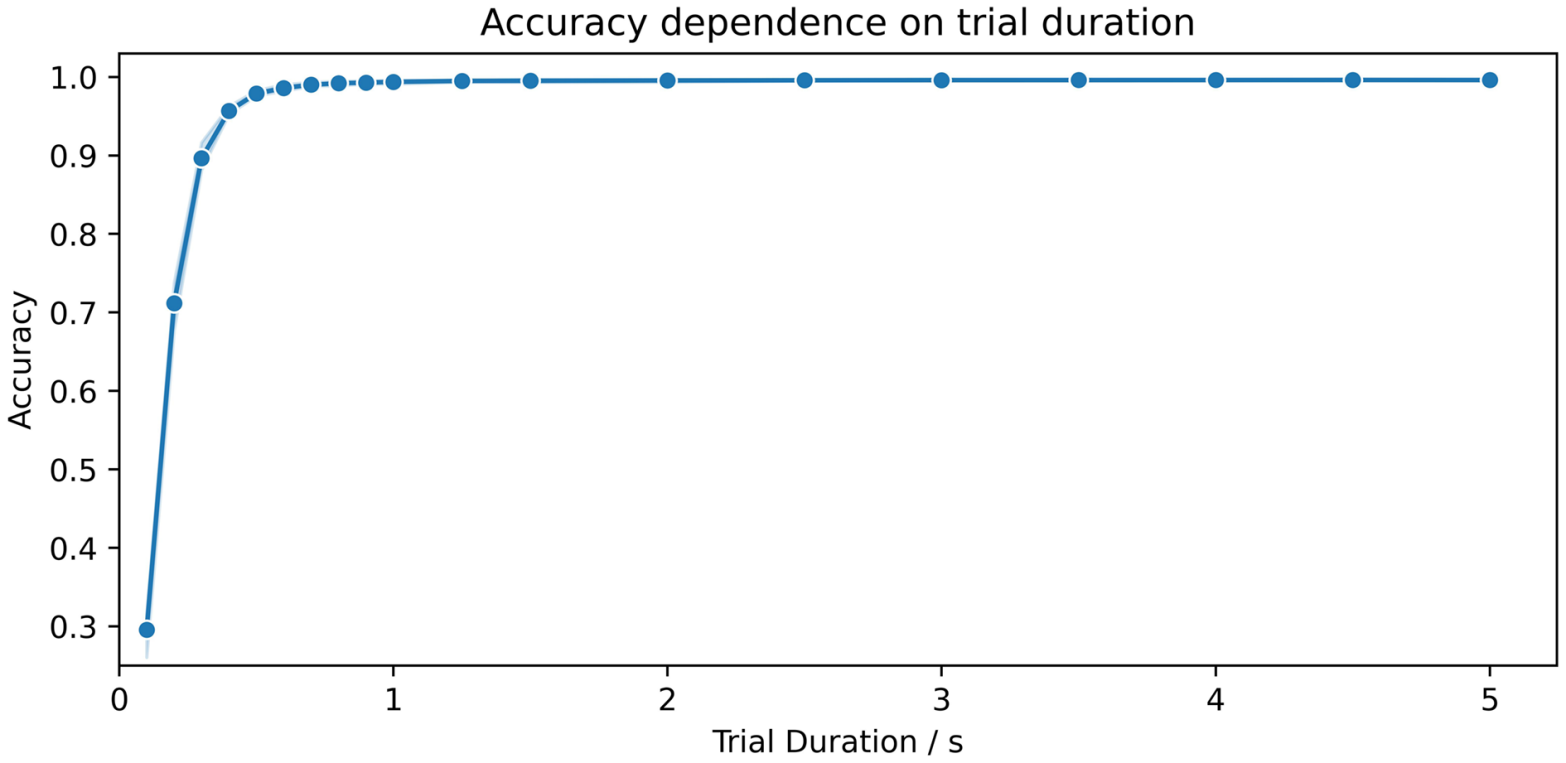
Dependence of the accuracy scores on the duration of the time segments. The number of kernels for the MiniRocket classifier was set to 3,500 and trained on 15 time segments. The duration of segments varied from 0.1 to 5 s. The blue shaded area indicates the upper and lower range of the classification accuracy between the 10 repetitions with a random selection of time segments and a random initialization of kernels.

The dependency of the accuracy scores on the trial duration is shown in Figure 3. For segment durations ranging from 0.1 s to 0.5 s, there is a sharp increase in accuracy, while for durations of 1 s in length, scores above 99.4% could already be achieved. Only a marginal increase in accuracy, from 99.5 to 99.6%, was achieved for durations ranging from 2.0 s to 5.0 s. A summary of all results and combinations is shown in Table 2.

The MiniRocket classification accuracy scores obtained through the LOOM method for neural fingerprinting based on resting-state data are as follows: The average accuracy score after cross-validation for training on rs1 and testing on rs2 was 99.15% ± 0.078%. Similarly, the mean Recall and Precision were found to be 99.15% ± 0.078 and 98.72% ± 0.425%, respectively, and the F1-Score was 98.80% ± 0.124% (Table 3). For training on rs2 and testing on rs1, the average accuracy score was found to be slightly larger with 99.96% ± 0.036%, as compared to the accuracy of 99.15% for training on rs1 and testing on rs2. This tendency was also observed in the other three metrics (cf. Table 3). Since the probability of obtaining a match for a single subject out of 124 subjects is 1/124, which is about 0.0081, the chance level in our experiment is approximately 0.81%. The difference in accuracy between the two classification tests were found to be significant, but with a change in score around the chance level (0.80–1.24%).

**Table 3:** Loom-based performance scores for two classification tests with the number of kernels set to 3,500, the number of trials to 15, and its duration to 1.5 s.

Metric	rs1 − rs2	rs2 − rs1
Accuracy	99.15 ± 0.0779	99.96 ± 0.0364
Precision	98.72 ± 0.4250	99.96 ± 0.0338
Recall	99.15 ± 0.0779	99.96 ± 0.0364
F1-Score	98.80 ± 0.1238	99.96 ± 0.0364

### 3.2. Influence of empty-room noise

Ten random trials were sampled per subject and per set with a trial duration of 1.5 s and 3,500 kernels. Whenever data originated from the same recordings, the continuous signal for each subject was split into two parts, and the trials for training and testing were sampled from the first and second half of the recording, respectively.

**Figure 4:**
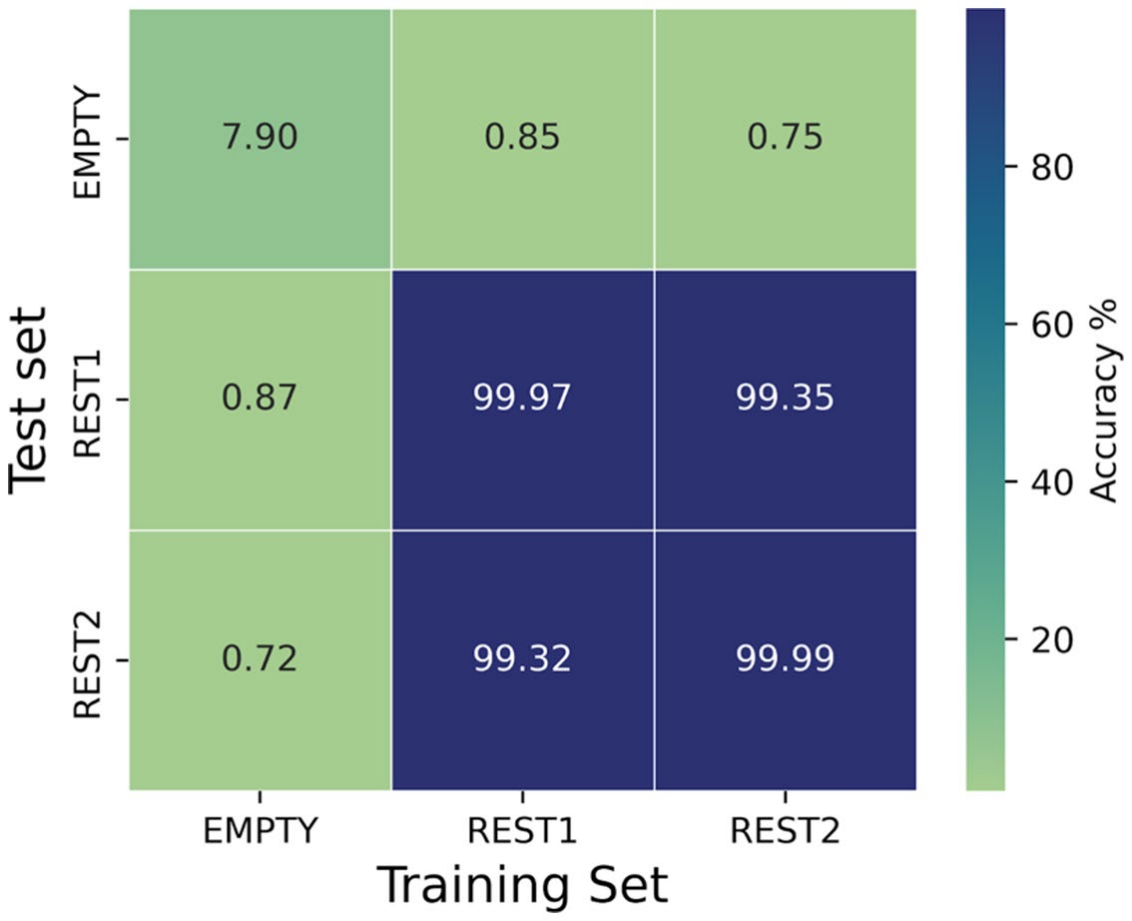
Dependence of the accuracy scores of the MiniRocket classifier on different combinations of training and test sets. Since rest1, rest2, and empty were all recorded on the same day, it is possible to isolate the contribution of the daily background noise to the classification outcomes. The number of kernels for the MiniRocket classifier was set to 3,500. For both the training and test set, 10 time segments of 1.5 s were sampled from each of the subjects.

Figure 4 shows the dependence of the MiniRocket classifier accuracy scores on different combinations of training and test sets. The results show that accuracies above 99.3% were achieved for all combinations of training and testing on resting-state data (rs1 vs. rs2 and rs2 vs. rs1). For resting-state recordings evaluated against the empty room recordings from the same day, the accuracies were close to the chance level, as depicted in Figure 4. In the case of empty vs. empty room recordings, the classifier achieved a low accuracy of 7.9%.

## 4. Discussion

There are many promising applications of multivariate time series classifications (MTSC) in medicine and neuroscience, including in the diagnosis of medical conditions, personalized treatment planning, and the development of brain-computer interfaces (BCIs). With this study, we have shown that it is possible to perform neural fingerprinting directly on MEG time series without performing feature engineering. This is, to the best of our knowledge, the first time that neural fingerprinting has been achieved based on magnetic field changes in single trials of MEG time series recordings without the need for a feature-based analysis. Furthermore, the MiniRocket approach used in the study required fewer data (shorter trials) for successful classification and also improved accuracy. For example, previous MEG publications reached MEG resting-state classification accuracies with trial lengths of 30 s in healthy controls of about 94.9–96.2% (da Silva Castanheira et al., 2021), and 94.5–98.2% at trial lengths of 8 s (Sareen et al., 2021). In contrast, MiniRocket analysis with 3,500 kernels achieved a classification accuracy of over 99% when training the model on as little as 15 s of data and testing it on 1 s time segments. These results demonstrate that substantially fewer data are needed for accurate classification in comparison with previous approaches that use MEG data in combination with connectivity measures (Demuru et al., 2017; da Silva Castanheira et al., 2021; Sareen et al., 2021) or data from electroencephalography (EEG) using EEG power spectra (Kong et al., 2019; Demuru and Fraschini, 2020).

In our parameter investigation, we aimed to explore the minimum input data requirements while maintaining computational efficiency. Our tests on trial duration suggested that a minimum of 0.9 s and 15 trials were sufficient to achieve accuracies above 99%. In terms of the number of trials, we found that training a MiniRocket classifier with 3,500 kernels requires at least nine trials of 1.5 s length to achieve accuracies above 99%. During our exploration of the number of kernels, we observed that increasing the number of kernels led to improved results in the low data regime, at the expense of computational demand. We were surprised to find that accuracies saturated at a relatively low number of 3,500 kernels using a fixed set of 15 trials of 1.5 s duration, resulting in accuracies above 99.3% (Table 2).

Interestingly, we observed a small but significant difference in all metrics when we reversed the order of training and evaluation set using the LOOM method. Specifically, the accuracies were 99.96% when training on rs1 and testing on rs2, whereas they fell to 99.15% when the order was reversed (Table 3). This difference in accuracy of 0.81% is about chance level and may be due to a single subject only. In principle, we did not expect the accuracies to be identical as the two measurements will not be identical in practice. The subject’s condition, such as mood and fatigue, is very likely to have an influence on the matching performance. Moreover, another source contributing to this difference may be due to a slight reduction in data quality over long recording sessions, possibly caused by increased subject movement due to fatigue or the execution of tasks before the second resting-state session. These findings raise the possibility that prioritizing training on datasets with higher complexity and diversity could be more crucial than employing the most complex data exclusively at the time of testing. However, in future work, it would be very interesting to investigate the model performance in a cohort of subjects where the temporal distance between rs1 and rs2 is increased by means of hours, days, weeks, and months.

In summary, these results are a proof of concept that subject differentiation can, in principle, be achieved directly from MEG brain recordings as short as 1 s to achieve high accuracies of about 99% using MiniRocket. This would greatly simplify current procedures as the technique does not require the selection of the best-performing feature for the classification model – as is the case when using functional connectomes (da Silva Castanheira et al., 2021; Sareen et al., 2021), for which the best-performing method needs to be determined. The high classification accuracy and the need for only relatively short segments of single trials data make MiniRocket a promising candidate for BCI research and motivate further research into the application of MiniRocket to MEG recordings.

### 4.1. Limitations

It has been suggested that day-to-day variations in the background noise during the recording may contribute significantly to the classification (da Silva Castanheira et al., 2021). We investigated this possibility by training the classifier on the subject’s recording and testing on corresponding empty-room data, which were recorded soon after the experiment. While our study shows that training the classifier on empty-room data and applying it to the subject’s resting-state data or vice versa did not result in the correct identification of individuals, and accuracies achieved on the cross-over of resting-state measurements and empty-room measurements were approximately at chance level, our findings suggest that the background noise may have a minor influence on the fingerprinting classification results. Notably, our analysis shows that matching empty room signals could be identified with an accuracy of approximately 8%.

To further investigate the classification performance and limitations on neural fingerprinting, we plan to implement a longitudinal study design to investigate the stability and performance of the classifier over time. Moreover, given that the subject is the class to be identified in this approach, we cannot split the data into training and test sets by subjects for the typical generalization purposes, which is a limitation of the method and is similar to a fingerprint analysis in criminal investigations, where a match can only be found if the suspect’s fingerprints are already in the database.

## Data availability statement

The original contributions presented in the study are included in the article/supplementary material, further inquiries can be directed to the corresponding author.

## Ethics statement

The studies involving humans were approved by the ethical committee of the RWTH Aachen University, Aachen, under the code EK 249/22. The studies were conducted in accordance with the local legislation and institutional requirements. The participants provided their written informed consent to participate in this study.

## Author contributions

NK and CK contributed equally to the conception and design of the study and to the data analysis. CK wrote the original draft. NS, IN, and JD supervised the study and acquired funding. All authors contributed to the article and approved the submitted version.

## Funding

Funded by the Deutsche Forschungsgemeinschaft (DFG, German Research Foundation) – 491111487, by the HBP SGA3 – Human Brain Project Specific Grant Agreement 3 (2020-04-01–2023-03-31), Helmholtz Metadata Collaboration (HMC), by the Deutsche Forschungsgemeinschaft (DFG, German Research Foundation) – 368482240/GRK2416, and by the Joint Lab “Supercomputing and Modeling for the Human Brain.

## Conflict of interest

The authors declare that the research was conducted in the absence of any commercial or financial relationships that could be construed as a potential conflict of interest.

## Publisher’s note

All claims expressed in this article are solely those of the authors and do not necessarily represent those of their affiliated organizations, or those of the publisher, the editors and the reviewers. Any product that may be evaluated in this article, or claim that may be made by its manufacturer, is not guaranteed or endorsed by the publisher.
